# Comparison of Five Conductivity Tensor Models and Image Reconstruction Methods Using MRI

**DOI:** 10.3390/molecules26185499

**Published:** 2021-09-10

**Authors:** Nitish Katoch, Bup-Kyung Choi, Ji-Ae Park, In-Ok Ko, Hyung-Joong Kim

**Affiliations:** 1 Department of Biomedical Engineering, Kyung Hee University, Seoul 02447, Korea; nitish@khu.ac.kr (N.K.); josh_bk@naver.com (B.-K.C.); 2 Division of Applied RI, Korea Institute of Radiological and Medical Science, Seoul 01812, Korea; inogi99@kirams.re.kr

**Keywords:** electrical conductivity, anisotropy, magnetic resonance imaging (MRI), diffusion tensor imaging (DTI), conductivity tensor imaging (CTI)

## Abstract

Imaging of the electrical conductivity distribution inside the human body has been investigated for numerous clinical applications. The conductivity tensors of biological tissue have been obtained from water diffusion tensors by applying several models, which may not cover the entire phenomenon. Recently, a new conductivity tensor imaging (CTI) method was developed through a combination of B1 mapping, and multi-b diffusion weighted imaging. In this study, we compared the most recent CTI method with the four existing models of conductivity tensors reconstruction. Two conductivity phantoms were designed to evaluate the accuracy of the models. Applied to five human brains, the conductivity tensors using the four existing models and CTI were imaged and compared with the values from the literature. The conductivity image of the phantoms by the CTI method showed relative errors between 1.10% and 5.26%. The images by the four models using DTI could not measure the effects of different ion concentrations subsequently due to *prior* information of the mean conductivity values. The conductivity tensor images obtained from five human brains through the CTI method were comparable to previously reported literature values. The images by the four methods using DTI were highly correlated with the diffusion tensor images, showing a coefficient of determination (R${}^{2}$) value of 0.65 to 1.00. However, the images by the CTI method were less correlated with the diffusion tensor images and exhibited an averaged R${}^{2}$ value of 0.51. The CTI method could handle the effects of different ion concentrations as well as mobilities and extracellular volume fractions by collecting and processing additional B1 map data. It is necessary to select an application-specific model taking into account the pros and cons of each model. Future studies are essential to confirm the usefulness of these conductivity tensor imaging methods in clinical applications, such as tumor characterization, EEG source imaging, and treatment planning for electrical stimulation.

## 1. Introduction

Electrical conductivity of biological tissues is determined by the cell density, extracellular volume fraction, composition and amount of extracellular matrix materials, and membrane characteristics as well as concentrations and mobility of ions in the extracellular and intracellular fluids [1]. The apparent macroscopic conductivity of such a composite material has been studied since the early 1900s and can be expressed as a weighted sum of conductivity values of its components based on the volume fractions and other factors [2,3]. The extracellular and intracellular fluids are conductors through which conductivity values are determined by concentrations and mobilities of ions and other mobile charge carriers. Cells with thin membranes behave like an insulator and lossy dielectric at low and high frequencies, respectively. Extracellular matrix materials are lossy dielectrics. Therefore, the macroscopic tissue conductivity exhibits frequency dependency [4,5].

When elongated cells are aligned towards a certain direction, movements of ions in the extracellular fluid are consequently hindered. Under a low-frequency electric field, the ions in the extracellular space are forced to move along the longitudinal direction, thereby making their mobilities direction-dependent. Therefore, in the white matter and muscle, the conductivity exhibits anisotropic properties at low frequencies. However, at high frequencies, the insulating cell membranes behave like a capacitor, and the anisotropic properties disappear above 1 MHz, for example [1,6]. In this paper, we approximately express the low-frequency conductivity of biological tissue as a tensor that is a symmetric positive definite 3 × 3 matrix [7,8]. At a high-frequency above 1 MHz, the conductivity is expressed as a scalar quantity [9].

Electrical conductivity is a passive material property whose measurement requires a probing current to generate a signal affected by the conductivity. In impedance imaging area, there are two different approaches in conductivity imaging using MRI. Magnetic resonance electrical impedance tomography (MREIT) reconstructs an image of low-frequency isotropic conductivity ($\sigma_{L}$) distribution by injecting low-frequency currents into a subject and measuring the induced magnetic flux density distributions using an MRI scanner [10,11,12]. Magnetic resonance electrical properties tomography (MREPT) produces high-frequency isotropic conductivity ($\sigma_{H}$) and permittivity ($\epsilon_{H}$) images by generating a radio-frequency (RF) eddy current that is affected by $\sigma_{H}$ and $\epsilon_{H}$ and measuring an induced RF magnetic field using a B1 mapping method [13,14,15].

In the diffusion tensor imaging (DTI) area, conductivity tensor image reconstructions have been investigated based on a physical relationship that conductivity and water diffusion tensors, denoted as C and D, respectively, have the same eigenvectors [16]. Based on this, Tuch et al. derived the first linear model between C and D, which enabled transformation of a diffusion tensor image into a conductivity tensor image [17,18]. Based on the idea of cross-property relation [17], three more conductivity tensor models and image reconstruction methods using DTI were developed [19,20,21,22]. These conductivity tensor models are based on a linear relation of C=ηD and the corresponding image reconstruction methods determine the scale factor η.

Although MREIT and MREPT reconstruct images of $\sigma_{L}$ and $\sigma_{H}$, respectively, through measurement of the effects of both ion concentrations and mobilities, neither of these two methods can produce an image of C. On the other hand, the four methods using DTI produce an image of C, but the effects of ion concentrations are not adequately demonstrated. To overcome these limitations, Sajib et al. developed a novel low-frequency conductivity tensor imaging (CTI) method [23]. In the first step of the CTI method, MREPT is used to reconstruct an image of $\sigma_{H}$ including the effects of both ion concentrations and mobilities at the Larmor frequency. In the second step, they apply a multi-b diffusion weighted imaging method to obtain an image of D and pixel-by-pixel information about extracellular and intracellular spaces. Combining all of these, the scale factor η between C and D is determined for every pixel using the following: (1) a physical relationship between the water diffusivity and ion mobility and (2) a model-based relation between $\sigma_{H}$ and $\sigma_{L}$ [6,24].

Recently, Wu et al. reviewed the DTI-based reconstruction models [25]. They reported that the conversion coefficient of water diffusion tensor to electrical conductivity tensor should require the information on ion concentration and extracellular volume fraction [25]. In addition, knowledge of accurate anisotropic conductivity can achieve reliable volume conduction models of electrical brain stimulation and EEG dipole reconstructions [26,27]. Over the years, considerable variation in the conductivity reconstruction models used for such approaches has been observed [27,28,29]. Consequently, an in-depth investigation of conductivity tensor models was required.

In this paper, we compared the accuracy of the five methods in reconstructed conductivity images of two phantoms using a 9.4 T research MRI scanner. The data acquired from five human subjects using a clinical 3 T MRI scanner were used to reconstruct conductivity tensor images of the brains using the five methods. The reconstructed conductivity tensor images of the human brains were analyzed and compared with each other and also with previous literature values.

## 2. Five Conductivity Tensor Models

### 2.1. Linear Eigenvalue Model (LEM)

Adopting the physical analysis that C and D have the same eigenvectors [16], Tuch et al. assumed that the conductivity of the intracellular space is negligible, that is, $\sigma_{i}\approx 0$ at low frequencies, and as a result derived the following relation between the eigenvalues of C and D [17]:

(1) $$c_{m}\approx \frac{\sigma_{e}}{d_{e}}\left[d_{m}\left(\frac{d_{i}}{3d_{e}}+1\right)+\frac{d_{m}^{2}d_{i}}{3d_{e}^{2}}-\frac{2}{3}d_{i}\right]$$

where $c_{m}$ and $d_{m}$ for m=1,2,3 are the eigenvalues of C and D, respectively; $\sigma_{e}$ is the extracellular conductivity; and $d_{i}$ and $d_{e}$ are the intracellular and extracellular water diffusion coefficients, respectively. Assuming that $d_{i}\approx 0$, the following linear relation between C and D was derived:

(2) $$C=\frac{\sigma_{e}}{d_{e}}D=\eta D.$$

Without measuring $\sigma_{e}$ and $d_{e}$ in (2), the empirically-calculated scale factor η=0.844 S·s/mm${}^{3}$ was applied to all pixels [17].

Since this empirical scale factor does not consider any intra-subject and inter-subject variabilities, a modified approach was proposed where the volume of the conductivity tensor ellipsoid was matched with the cubed value of the isotropic conductivity using the least square method [18]. For the human brain, the scale factor η was determined as

(3) $$\eta =\frac{d_{WM}\sigma_{WM}+d_{GM}\sigma_{GM}}{{d_{WM}}^{2}+{d_{GM}}^{2}}$$

where $d_{WM}$ and $d_{GM}$ are the measured water diffusion coefficient of the white matter (WM) and gray matter (GM), respectively. In this paper, we used (3) with the literature values of $\sigma_{WM,GM}$ = 0.14 and 0.27 S/m, respectively, for human brain imaging experiments [30,31,32]. For a conductivity phantom, $\sigma_{WM}$ and $\sigma_{GM}$ were replaced by measured conductivity values using an impedance analyzer.

### 2.2. Force Equilibrium Model (FEM)

The relation between the diffusion coefficient *d* and viscosity ν is given by the Stokes–Einstein relation as follows:

(4) $$d=\frac{k_{B}T}{6\pi \nu r}$$

where $k_{B}$ is Boltzmann’s constant, *T* is the absolute temperature, and *r* is the radius of a spherical particle [19]. From the equilibrium condition between the electric and viscous forces, the conductivity σ can be expressed as

(5) $$\sigma =\frac{J}{E}=\frac{q^{2}N}{6\pi r\nu }$$

where *J* and *E* are the magnitude of the current density and electric field, respectively, $q=1.6\times 10^{-19}$ C and $N=2\times 10^{25}$ m${}^{-3}$. Applying (4) and (5) to the extracellular space, the following relation can be derived:

(6) $$\sigma_{e}=\frac{0.76q^{2}N}{k_{B}T}d_{e}$$

where $k_{B}T$ = $4.1\times 10^{-21}$ J. For an anisotropic case, Sekino et al. assumed that the following relation can be inferred from (6) [19]:

(7) $$C=\frac{0.76q^{2}N}{k_{B}T}D_{e}=\eta D_{e}.$$

To measure $D_{e}$ in (7) for each pixel, Sekino et al. [20] adopted the following bi-exponential model for a diffusion-weighted MRI signal $S_{b}$ with a given *b* value [33,34]:

(8) $$\frac{S_{i}\left(b\right)}{S_{i}\left(0\right)}=v_{f,i}e^{-bd_{f,i}}+v_{s,i}e^{-bd_{s,i}}$$

where $S_{i}\left(b\right)$ denote the MR signal with a diffusion gradient (b≠0) along the *i*th direction, $S_{i}\left(0\right)$ is the MRI signal without applying a diffusion gradient (b=0), $v_{f,i}$ and $d_{f,i}$ are the volume fraction and diffusion coefficient of a fast diffusion component along the *i*th direction, respectively, and $v_{s,i}$ and $d_{s,i}$ are the volume fraction and diffusion coefficient of a slow diffusion component along the *i*th direction, respectively. Using a curve fitting method, the fast diffusion tensor $D_{f}$ is extracted from the measured data of $S_{i}\left(b\right)$ and $S_{i}\left(0\right)$ in three orthogonal directions. In (7), $D_{e}$ is replaced by $D_{f}$ assuming that the fast diffusion corresponds to the extracellular diffusion.

### 2.3. Volume Constraint Model (VCM)

Miranda et al. [21] suggested a method to determine the scaling factor η at each pixel using the measured water diffusion tensor D and the values of equivalent isotropic conductivity $\sigma_{iso}$ for different brain tissues from the given literature [30,31,32,35] as follows:

(9) $$C=\frac{3\sigma_{iso}}{trace(D)}D=\eta D$$

where trace(D) is the sum of the three eigenvalues of D. In this paper, the conductivity phantom, $\sigma_{iso}$ was replaced by a measured conductivity value using an impedance analyzer. For the human brain, we used the literature values of $\sigma_{iso}$ = 0.14, 0.27, and 1.79 S/m for the WM, GM, and cerebrospinal fluid (CSF), respectively, [30,31,32,35].

### 2.4. Volume Fraction Model (VFM)

For the brain tissue, Wang et al. assumed that movements of water molecules and ions are constrained by its multi-compartment environment including axons, glial cells, and CSF [22]. The measured diffusion tensor D was expressed as

(10) $$D=S\;diag\left(d_{l},d_{t1},d_{t2}\right)\;S^{T}$$

where $d_{l},d_{t1}$, and $d_{t2}$ are the eigenvalues of D along the longitudinal and two transversal directions, respectively. Although the WM may include myelinated axons with various directions, the researchers assumed that three groups of the WM exist with their longitudinal directions in parallel to the longitudinal and two transversal directions of the measured diffusion tensor D. In addition, they assumed that the volume fractions of the three WM groups and the remaining isotropic tissues are $\alpha_{l},\alpha_{t1},\alpha_{t2}$, and $\alpha_{iso}$. Therefore, for this multi-compartment model, the following equations can be obtained:

(11) $$\left\{\begin{array}{c} \alpha_{l}e^{-bd_{l}^{W}}+\alpha_{t1}e^{-bd_{t1}^{W}}+\alpha_{t2}e^{-bd_{t2}^{W}}+\left(1-\alpha_{l}-\alpha_{t1}-\alpha_{t2}\right)e^{-bd_{iso}}=e^{-bd_{l}}\\ \alpha_{l}e^{-bd_{t1}^{W}}+\alpha_{t1}e^{-bd_{l}^{W}}+\alpha_{t2}e^{-bd_{t2}^{W}}+\left(1-\alpha_{l}-\alpha_{t1}-\alpha_{t2}\right)e^{-bd_{iso}}=e^{-bd_{t1}}\\ \alpha_{l}e^{-bd_{t2}^{W}}+\alpha_{t1}e^{-bd_{t1}^{W}}+\alpha_{t2}e^{-bd_{l}^{W}}+\left(1-\alpha_{l}-\alpha_{t1}-\alpha_{t2}\right)e^{-bd_{iso}}=e^{-bd_{t2}} \end{array}\right.$$

where $d_{iso}$ is the diffusion coefficient of the isotropic tissues; $d_{l}^{W},d_{t1}^{W}$, and $d_{t2}^{W}$ are the diffusion coefficients of the WM in the longitudinal and two transversal directions, respectively; and $\alpha_{l}+\alpha_{t1}+\alpha_{t2}+\alpha_{iso}=1$.

The eigenvalues of the conductivity tensor C are expressed as a weighted sum of the conductivity values of the four compartments as:

(12) $$\left\{\begin{array}{c} \sigma_{l}=\alpha_{l}\sigma_{l}^{W}+\alpha_{t1}\sigma_{t1}^{W}+\alpha_{t2}\sigma_{t2}^{W}+\alpha_{iso}\sigma_{iso}\\ \sigma_{t1}=\alpha_{l}\sigma_{t1}^{W}+\alpha_{t1}\sigma_{l}^{W}+\alpha_{t2}\sigma_{t2}^{W}+\alpha_{iso}\sigma_{iso}\\ \sigma_{t2}=\alpha_{l}\sigma_{t2}^{W}+\alpha_{t1}\sigma_{t1}^{W}+\alpha_{t2}\sigma_{l}^{W}+\alpha_{iso}\sigma_{iso} \end{array}\right.$$

where $\sigma_{l}^{W}$, $\sigma_{t1}^{W}$, and $\sigma_{t2}^{W}$ are the conductivity values of the WM in the longitudinal and two transversal directions, respectively. The values of $\sigma_{l}^{W}$, $\sigma_{t1}^{W},\sigma_{t2}^{W}$, and $\sigma_{iso}$ are not measured; instead, the literature values are adopted [32]. Note that the VFM method does not use the scale factor η and can be used only for the brain tissue including the WM. For the GM and CSF, isotropic conductivity values from existing literature are used. The constraint of $\sigma_{t1}=\sigma_{t2}$ is applied in the implementation of the VFM method.

### 2.5. Conductivity Tensor Imaging (CTI) Model

The CTI method derives a low-frequency conductivity tensor C by using a high-frequency isotropic conductivity $\sigma_{H}$ obtained using the MREPT technique and the information about water diffusion obtained by the multi-b diffusion weighted imaging method. Since the details of its basic theory and algorithm are available in [6,23,24], we simply introduce the following CTI formula in this paper:

Here, the low-frequency conductivity tensor is expressed as

(13) $$C=\alpha {\bar{c}}_{e}D_{e}$$

Using the reference value of β=0.41 [23,24], the apparent extracellular ion concentration (${\bar{c}}_{e}$) of (13) can be estimated as

(14) $${\bar{c}}_{e}=\frac{\sigma_{H}}{\alpha d_{e}^{w}+\left(1-\alpha \right)d_{i}^{w}\beta }$$

Using ${\bar{c}}_{e}$ from (14) in (13), low-frequency conductivity tensor can be expressed as

(15) $$C=\alpha {\bar{c}}_{e}D_{e}=\frac{\alpha \sigma_{H}}{\alpha d_{e}^{w}+\left(1-\alpha \right)d_{i}^{w}\beta }D_{e}=\eta D_{e}$$

where $\sigma_{H}$ is the high-frequency conductivity at the Larmor frequency, α is the extracellular volume fraction, ${\bar{c}}_{e}$ is apparent extracellular ion concentration, β is the ion concentration ratio of intracellular and extracellular spaces, $d_{e}^{w}$ and $d_{i}^{w}$ are the extracellular and intracellular water diffusion coefficients, respectively, and $D_{e}$ is the extracellular water diffusion tensor.

## 3. Imaging Experiments and Data Processing

### 3.1. Phantom Imaging

To compare the accuracy of the reconstructed conductivity images based on five methods described in Section 2, we used two conductivity phantoms, each with three compartments of known conductivity values. The compartments were filled with electrolytes or giant vesicle suspensions. The giant vesicles were cell-like materials with thin insulating membranes [36].

Phantom #1 comprised of two compartments of electrolytes (EL${}_{1}$ and EL${}_{2}$) and one compartment of a giant vesicle suspension (GVS${}_{1}$) where giant vesicles were suspended in the electrolyte EL${}_{1}$. Phantom #2 comprised of two compartments of different electrolytes (EL${}_{3}$ and EL${}_{4}$) and one compartment of a different giant vesicle suspension (GVS${}_{2}$). Table 1 shows the concentrations of NaCl and CuSO${}_{4}$, the extracellular volume fraction, mobility, and low-frequency conductivity values, which are measured by an impedance analyzer (SI1260A, AMETEK, West Sussex, UK) at 10 Hz. For the electrolyte EL${}_{3}$, we increased its viscosity by adding 2 g/L of hyaluronic acid and 10 g/L of polyethylene glycol (PEG, average Mv 8000) solution, thereby decreased the ion mobility and also its conductivity value. In the giant vesicle suspension GVS${}_{2}$, we used electrolyte EL${}_{3}$.

A 9.4 T research MRI scanner (Agilent Technologies, Santa Clara, CA, USA) equipped with a single-channel birdcage coil (Model: V-HQS-094-00638-029, RAPID Biomedical GmbH, Rimpar, Germany) with a cubic voxel having 0.5 mm edge length was used for phantom imaging. For multi-b diffusion weighted imaging, we used the single-shot spin-echo echo-planar imaging (SS-SE-EPI) pulse sequence. The imaging parameters were as follows: repetition time (TR)/echo time (TE) = 2000/70 ms, number of signal acquisitions = 2, field-of-view (FOV) = 65 × 65 mm${}^{2}$, slice thickness = 0.5 mm, flip angle = 90${}^{\circ }$, and image matrix size = 128 × 128. The number of diffusion-weighting gradient directions was 30 with b-values of 50, 150, 300, 500, 700, 1000, 1400, 1800, 2200, 2600, 3000, 3600, 4000, 4500, and 5000 s/mm${}^{2}$. For high-frequency conductivity image reconstructions in the CTI method, B1 phase maps were acquired using the multi-slice multi-echo spin-echo (MS-ME-SE) pulse sequence. The imaging parameters were as follows: TR/TE = 2200/22 ms, number of signal acquisitions = 5, FOV = 65 × 65 mm${}^{2}$, slice thickness = 0.5 mm, flip angle = 90${}^{\circ }$, and image matrix size = 128 × 128. More details of the phantom preparation and data acquisition are described in [6,24].

### 3.2. In Vivo Human Imaging

Five healthy volunteers were recruited based on the protocol approved by the Institutional Review Board (IRB) at Kyung Hee University (KHSIRB-18-073). Informed consent forms were obtained from the volunteers before conducting the studies. In vivo human brain imaging experiments were performed using a clinical 3 T MRI scanner (Magnetom Trio A Tim, Siemens Medical Solution, Erlangen, Germany) with body coil in transmitting mode and an 8-channel head coil in receiving mode (3D head matrix, A Tim Coil, Siemens Medical Solution, Erlangen, Germany).

For multi-b diffusion weighted imaging, we used the SS-SE-EPI pulse sequence. The number of diffusion-weighting gradient directions was 15 with similar b-values used in the phantom experiments. The imaging parameters were as follows: TR/TE = 2000/70 ms, slice thickness = 4 mm, flip angle = 90${}^{\circ }$, number of averaging = 2, number of slices = 5, and acquisition matrix = 64 × 64. The matrix size of 64 × 64 was extended to 128 × 128 for subsequent data processing steps. The imaging time was 23 min for the multi-b diffusion data acquisition. For B1 mapping, the MS-ME-SE pulse sequence was used. The imaging parameters were as follows: TR/TE = 1500/15 ms, number of echoes = 6, number of averaging = 5, slice thickness = 4 mm, number of slices = 5, acquisition matrix = 128 × 128, and FOV = 240 × 240 mm${}^{2}$ using a scan duration of 16 min. For anatomical reference, a conventional T${}_{2}$-weighted scan was obtained. The total imaging time for each human subject was about 41 min. More details of the in vivo scans are described in [24].

### 3.3. Data Processing

The acquired multi-b diffusion data were preprocessed using the MRtrix3 (www.mrtrix.org, accessed on 10 March 2021) [37] and FMRIB software library (FSL, www.fmrib.ox.ac.uk/fsl, accessed on 10 March 2021) [38]. The preprocessing steps included MP-PCA denoising [39], Gibbs-ringing correction [40], and eddy current distortion correction [38]. The averaged images at b=0 were linearly coregistered to the T${}_{2}$-weighted images using the FLIRT method, and the affine transformation matrix was used to nonlinearly coregister the diffusion weighted images at b≠0 to the T${}_{2}$-weighted images using the FNIRT method [38]. The b-value of 700 s/mm${}^{2}$ was used for diffusion tensor image reconstructions in the LEM, VCM, and VFM methods. In the FEM and CTI methods, we calculated $D_{f}$ and $D_{s}$ using a bi-exponential model [24,34].

For high-frequency conductivity image reconstructions in CTI method, the acquired phase map data were first corrected for Gibbs-ringing artifacts [40], and then multi-channel multiple echoes were combined to achieve the best signal-to-noise ratio (SNR) [41,42]. The high-frequency conductivity images were reconstructed using the method proposed by Gurler et al. [14] in order to suppress boundary artifacts.

For human brain images, T${}_{2}$-weighted images were segmented into the WM, GM, and CSF regions using the MICO [43]. The summary of numbers of pixels in the regions of interest (ROIs) are given in Table 2. In each ROI, we excluded the outermost layer of two-pixel width to reduce partial volume effects. All the data processing steps were implemented using the MATLAB software (Mathworks, Natick, MA, USA). For the image reconstructions using the CTI method, we used the MRCI toolbox (https://iirc.khu.ac.kr/toolbox.html, accessed on 10 March 2021) [44].

## 4. Results

### 4.1. Two Phantoms

**Figure 1:**
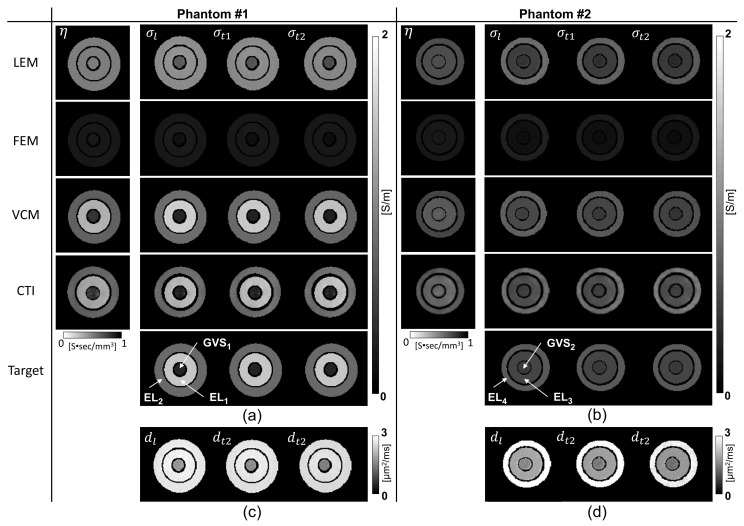
Reconstructed conductivity tensor images of phantoms #1 (**a**) and #2 (**b**). Target images are the true conductivity tensor images generated using the conductivity values measured by the impedance analyzer. (**c**,**d**) are the diffusion tensor images of phantoms #1 and #2, respectively. The VFM method was not applicable to the phantoms since it was specifically designed for the brain tissues.

**Figure 2:**
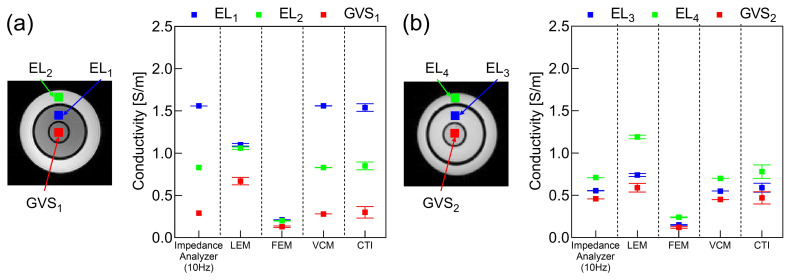
Values of $\sigma_{L}=\frac{\sigma_{l}+\sigma_{t1}+\sigma_{t2}}{3}$ in the reconstructed conductivity tensor images of phantoms #1 (**a**) and #2 (**b**). The values were compared with the conductivity values measured at 10 Hz using the impedance analyzer. The square symbol indicates the mean value and the bar represents the SD.

Figure 1 shows the reconstructed conductivity tensor and diffusion tensor images of phantoms #1 and #2. For both tensors, we plotted their longitudinal and two transversal components. The images of the scale factor η between two tensors were also plotted. Note that the VFM method could not be used for the phantoms since it is only applicable to an anisotropic object. The ROIs were defined as shown in Figure 2a,b corresponding to the three different compartments of phantoms #1 and #2, respectively. The mean and standard deviation (SD) of the reconstructed conductivity values for each ROI were calculated and compared with those that were independently measured using the impedance analyzer at 10 Hz as in Figure 2.

In the case of phantom #1 shown in Figure 1a and Figure 2a, the electrolytes EL${}_{1}$ and EL${}_{2}$ had different NaCl concentrations as shown in Table 1. The LEM and FEM methods failed to distinguish the difference in concentration, and the VCM and CTI methods were able to differentiate the difference. In phantom #2 shown in Figure 1b and Figure 2b, the conductivity of EL${}_{3}$ was reduced compared to that of EL${}_{4}$ due to the reduced mobility in EL${}_{3}$ as shown in Table 1. Since the diffusion coefficient was altered by this change in mobility, all four methods could distinguish the difference between EL${}_{3}$ and EL${}_{4}$ with the same NaCl concentration in phantom #2. Only the CTI method was able to measure the changes in both concentration and mobility without using *prior* information of the conductivity values measured by the impedance analyzer.

Table 3 shows the errors in the reconstructed conductivity values with respect to the reference values measured by the impedance analyzer (SI1260A, AMETEK Inc., Berwyn, PA, USA). Since the measured conductivity values using the impedance analyzer were used in place of $\sigma_{WM,GM,CSF}$ in (3), the values of η in the LEM method were different from each other for two phantoms. However, the LEM method could not account for the effects of the concentration difference in phantom #1 despite being able to detect the mobility difference in phantom #2. In the VCM method, the errors in the four electrolyte regions were zero since the measured conductivity values were used in place of $\sigma_{iso}$ in (9). The CTI method could recover the conductivity values with an error of 1.10% to 5.26% in all the regions of the electrolytes and giant vesicle suspensions.

### 4.2. Five Human Brains

**Figure 3:**
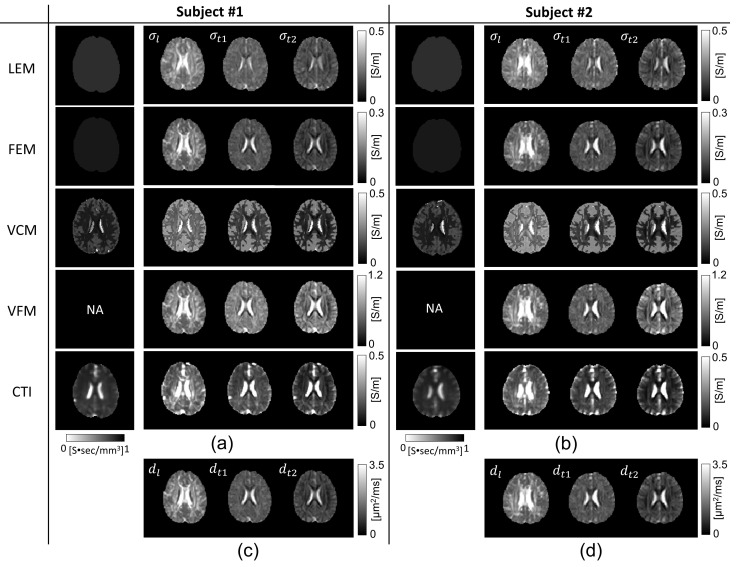
(**a**,**b**) are the longitudinal ($\sigma_{l}$) and transversal ($\sigma_{t1}$ and $\sigma_{t2}$) components of the reconstructed conductivity tensor images of the human brains from subjects #1 and #2, respectively, using the five methods of LEM, FEM, VCM, VFM, and CTI. (**c**,**d**) show the images of the water diffusion tensors.

**Figure 4:**
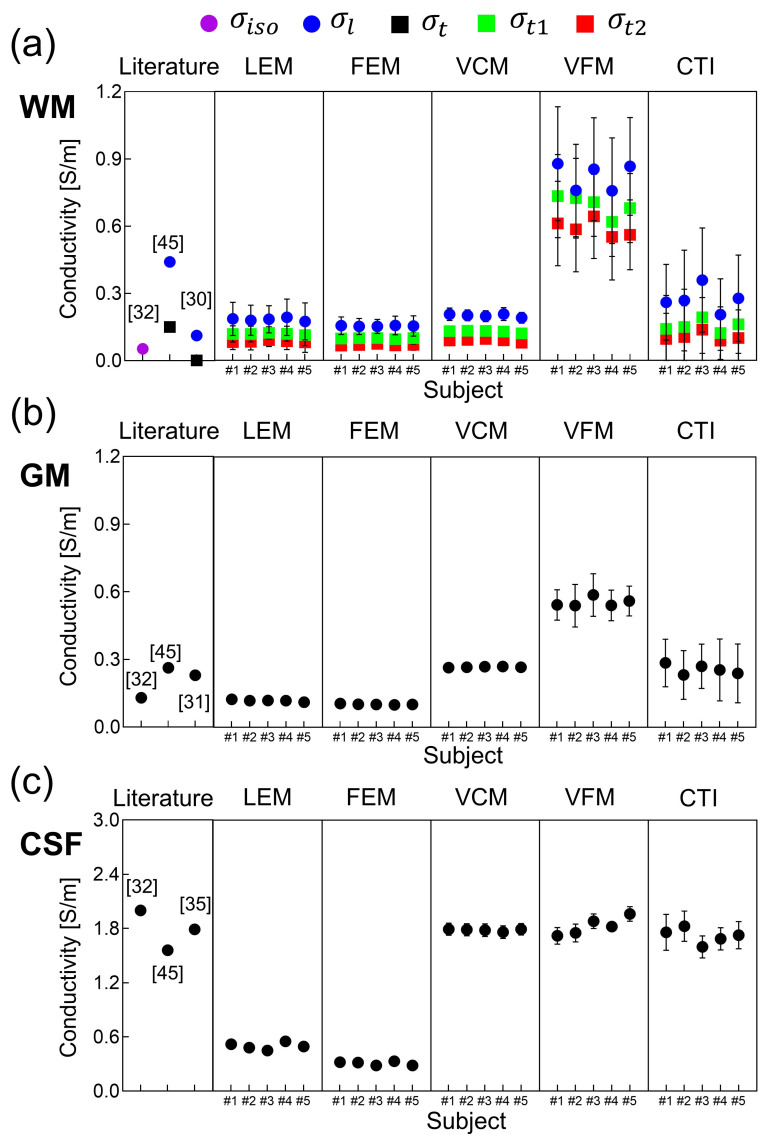
Mean and SD values of the reconstructed conductivity tensors using the five methods for all five human subjects are compared with the existing literature values. (**a**) are conductivity values from WM region, (**b**,**c**) are from GM and CSF, respectively. For the WM region, the mean and SD values of $\sigma_{l},\sigma_{t1}$, and $\sigma_{t2}$ are plotted. For the GM and CSF regions, the mean and SD values of $\sigma_{L}=\frac{\sigma_{l}+\sigma_{t1}+\sigma_{t2}}{3}$ are plotted. The vertical bar represents the SD.

Figure 3 shows the images of the longitudinal ($\sigma_{l}$) and transversal ($\sigma_{t1}$ and $\sigma_{t2}$) components of the reconstructed conductivity tensor images of the brains of the first and second subject using the five methods. Figure 4 shows the mean and SD values of the reconstructed conductivity tensors in the WM, GM, and CSF regions, which are summarized in Table 2 for all five subjects. For the WM region, the mean and SD values of $\sigma_{l},\sigma_{t1}$, and $\sigma_{t2}$ were plotted for the five different methods. For the GM and CSF regions, we plotted the mean and SD values of $\sigma_{L}=\frac{\sigma_{l}+\sigma_{t1}+\sigma_{t2}}{3}$.

In Figure 4, the LEM and FEM methods underestimated the conductivity values of the CSF region compared with the literature values [35]. For the WM and GM regions, the VFM method overestimated conductivity values compared to the literature values [30,31,32,45]. The conductivity values observed in the VCM method, especially for GM and CSF regions, were comparable to the literature [31,32,45]. Nevertheless, in the LEM and VCM method, we used the literature values of $\sigma_{iso}$ for the WM, GM, and CSF regions for all five subjects, and this resulted in a small amount of inter-subject variability. The CTI method produced conductivity values that were comparable to the existing literature values without using *prior* information of the literature values. In the WM region, the value of $\sigma_{l}$ obtained by the CTI method was between 0.19 and 0.32 S/m and the values of $\sigma_{t1}$ and $\sigma_{t2}$ were between 0.07 and 0.19 S/m, respectively. In the GM region, the value of $\sigma_{L}$ was between 0.23 and 0.30 S/m. The value of $\sigma_{L}$ in the CSF region was between 1.59 and 1.82 S/m with the mean value of 1.72 S/m for all the five subjects, consistent with those found in the existing literature [35,45].

**Figure 5:**
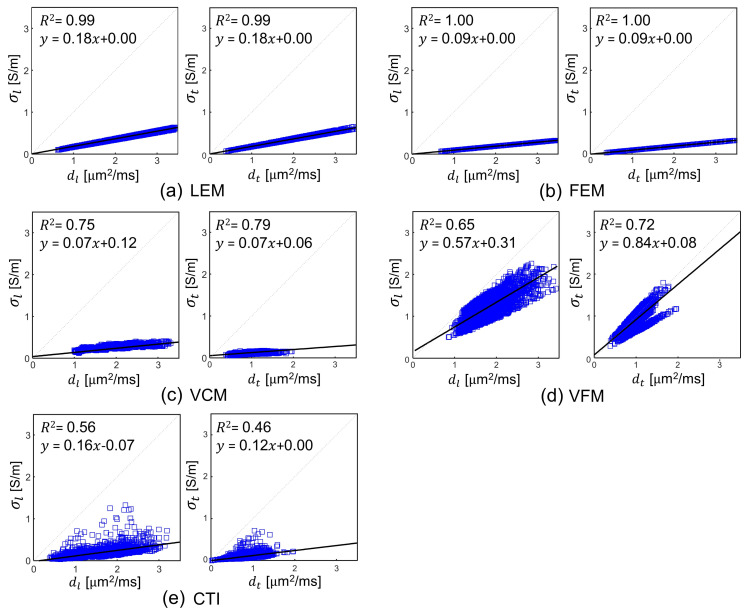
Results of the linear regression analyses between $s_{l}$ and $d_{l}$, the longitudinal component of both tensors and averaged transversal components $\sigma_{t}=\frac{\sigma_{t1}+\sigma_{t2}}{2}$ and $d_{t}=\frac{d_{t1}+d_{t2}}{2}$ in the reconstructed conductivity and water diffusion tensor images of all five human subjects pooled together.

We performed a correlation analyses to visualize the simultaneous influence of water diffusion tensor in reconstructed conductivity tensor. Figure 5 shows the plots of the linear regression analyses between $\sigma_{l}$ and $d_{l}$, the longitudinal component of both tensors and averaged transversal components $\sigma_{t}=\left(\sigma_{t1}+\sigma_{t2}\right)/2$ and $d_{t}=\left(d_{t1}+d_{t2}\right)/2$ for the WM, GM, and CSF regions in all five human subjects (Appendix A). For the LEM and FEM methods, the coefficient of determination ($R^{2}$) was 0.99 and 1.00, respectively. Therefore, these two methods, may not provide additional information that is not available in the water diffusion tensor. For the VCM and VFM methods, $R^{2}$ ranges from 0.65 to 0.79, respectively, whereas the CTI method showed lesser correlation between $\sigma_{l,t}$ and $d_{l,t}$ with $R^{2}$ ranges from 0.46 and 0.56. In the CTI method, the magnitudes of C and D provided moderately dependent information although they have the same directional property, i.e., the same eigenvectors. This is also because the conductivity tensor in the CTI method has a predominant effect of apparent ion concentrations.

To analyze the directional property, we also computed the anisotropy ratio (AR) in the WM region expressed as $AR_{C}=\frac{2\sigma_{l}}{\sigma_{t1}+\sigma_{t2}}$ and $AR_{D}=\frac{2d_{l}}{d_{t1}+d_{t2}}$ for the conductivity tensor and diffusion tensor, respectively [46]. The mean and SD value of $AR_{D}$ were 2.52 ± 0.76, whereas the mean and SD values of $AR_{C}$ were 2.53 ± 0.77, 2.52 ± 0.78, 2.50 ± 0.78, 1.73 ± 0.47, and 2.52 ± 0.84 for the LEM, FEM, VCM, VFM, and CTI methods, respectively, for the five human subjects. The lower value of $AR_{C}$ in the VFM model can be attributed to the assumption of $\sigma_{t1}=\sigma_{t2}$. For the other four methods, the values of $AR_{C}$ and $AR_{D}$ were similar since the structural property primarily determined the anisotropy.

**Figure 6:**
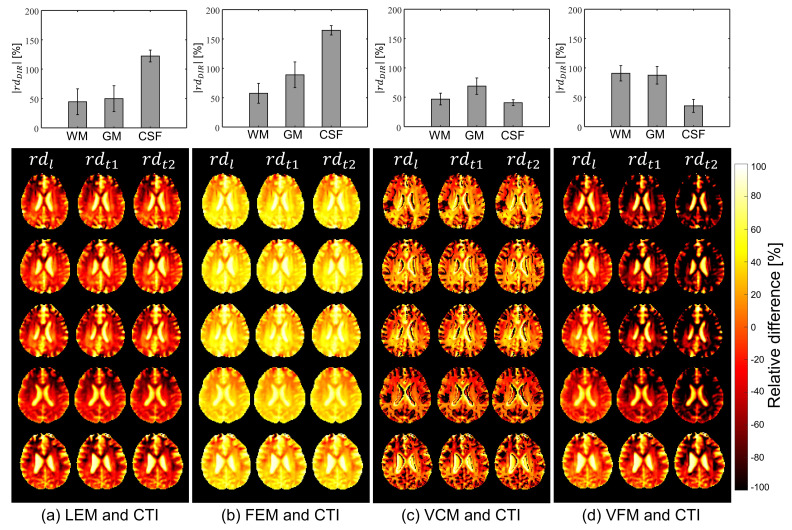
Images of the relative differences in the longitudinal ($e_{l}$) and transversal ($e_{t1}$ and $e_{t2}$) components between one of the LEM, FEM, VCM, and VFM methods and the CTI method. The graphs at the top are the absolute mean and SD values of the relative differences ($|rd_{DIR}|$) in the WM, GM, and CSF regions. The bar represents the SD.

Using the CTI method as a reference, we plotted the images of the relative difference ($rd_{DIR}$), which are defined as

(16) $$rd_{DIR}=\frac{\sigma_{DIR,CTI}-\sigma_{DIR,MTH}}{\sigma_{DIR,CTI}}\times 100\;\left(\%\right)$$

where DIR is l,t1, or t2 and $\sigma_{DIR,CTI}$ and $\sigma_{DIR,MTH}$ are the reconstructed $\sigma_{l},\sigma_{t1}$, or $\sigma_{t2}$ using the CTI method and one of the LEM, FEM, VCM, and VFM methods, respectively (Appendix A). Figure 6 shows the images of $rd_{DIR}$ for all five subjects pooled together. Table 4 summarizes the absolute mean values of $|rd_{DIR}|$ for the WM, GM, and CSF regions for each subject. The bar plots at top of Figure 6 show absolute mean values of $|rd_{DIR}|$ for all five subjects for WM, GM, and CSF regions, and images at the bottom of Figure 6 show the relative difference with sign of deviation to CTI method. The mean and SD of the relative difference for the entire brain slice of the five subjects were 69.63 ± 31.11, 104.94 ± 35.41, 53.35 ± 25.15, and 68.83 ± 14.38% for the LEM, FEM, VCM, and VFM methods, respectively.

## 5. Discussion

In this paper, we compared five conductivity tensor models and image reconstruction methods using two phantoms and five human subjects. The data from the phantom experiments were used to evaluate the accuracy of the reconstructed conductivity images against the measured conductivity values obtained using the impedance analyzer. In vivo human experiment data were used to compare the values of the reconstructed conductivity tensors with existing literature values and also with each other using the CTI method as a reference.

In the phantom experiments, the LEM and FEM methods could not distinguish the electrolytes with different NaCl concentrations. The errors in the reconstructed conductivity images using the LEM and FEM were relatively large. The errors of the VCM method were the smallest since we used the conductivity values measured using the impedance analyzer in place of $\sigma_{iso}$ in (9). In most clinical applications where the value of $\sigma_{iso}$ is unknown, the VCM method may fail to recover the inhomogeneous conductivity values. In addition, living tissues are heterogeneous, and their conductivity varies with the pathophysiology condition. Thus, a single global value of conductivity to the entire tissue type may not sufficient. The VFM method could not be applied to the phantoms since it was explicitly designed for anisotropic brain tissues. Without relying on *prior* information of mean isotropic conductivity values, the CTI method recovered conductivity values with an error ranging from 1.10% to 5.26% for all six different compartments in the two phantoms having different ion concentrations and mobilities. Furthermore, the CTI method could properly handle the effects of different cell densities in two giant vesicle suspensions with different extracellular volume fractions.

Using the data from in vivo human brain imaging experiments, we produced conductivity tensor images of the brains as shown in Figure 3 using the five methods. Compared to the conductivity values of the WM, GM, and CSF from existing literature, the values using the LEM and FEM methods had large amounts of bias in the WM and GM regions. Since the global scale factor was used for all pixels in the FEM method, the method’s ability to handle intra-subject and inter-subject variability appeared to be primarily limited. Although the scale factor η in the LEM method contains inter-subject variability, but still lacks intra-subject tissue heterogeneity. Furthermore, the global scale factor does not account for position dependence, and its deviation in the tissues only explains the water diffusion alterations. The conductivity values in CSF from both LEM and FEM methods were significantly underestimated compared to the literature values. The calculated conductivity tensor in both these methods was simply linearly scaled water diffusion tensor, and hence may explain their considerable low conductivity measurements [17,19]. The conductivity values measured in our study were matched with previous studies by Rullman et al. and Sekino et al. [18,20].

In the volume constrained method (VCM), conductivity values of GM and CSF were matched with the literature and also with the CTI method in our study. In contrast, conductivity values of WM were underestimated at longitudinal directions. This might be due to the use of similar literature values of $\sigma_{iso}$ in all three fiber directions. This method still lacks the intra-voxel heterogenic property of the tissues, and segmentation inaccuracies may end up assigning incorrect $\sigma_{iso}$ values. An introduction of the isotropic extracellular electrical conductivity from MREIT could be helpful to overcome this limitation [12]. In the volume fraction method (VFM), conductivity values in WM and GM were highly overestimated. This stems from the fact that the eigenvalues of conductivity tensor were computed as a weighted sum of conductivity values associated with four compartments of individual fiber bundle direction. VFM method is computationally complicated, and the ill-posed problem may lead to failed fit in noisy voxels [22]. In addition, the assumption of constant $\sigma_{L}$ and $\sigma_{t1}=\sigma_{t2}$, which were then set to be literature conductivity value, somehow neglected the intra-voxel heterogeneity, and reduced the anisotropic ratio. Instead of such limitations, VFM method effectively handled the partial volume effect.

Although the scale factor η in the LEM, FEM, and VCM methods was computed for each pixel, their ability to handle the intra-subject and inter-subject variability was also limited as a result of fixed literature conductivity values of the WM, GM, and CSF regions for all subjects. The VFM method manages cross-subject variability but failed with intra-subject flexibility. In contrast to the four methods using DTI only, the CTI method produced conductivity tensor images with values that were comparable to existing literature values. Without using *prior* information about tissue conductivity values, the CTI method appeared to properly handle the intra-subject and inter-subject variability. However, the intra-subject and inter-subject variabilities, may have been affected in all methods by measurement noise, and systematic artifacts.

The recovered conductivity tensors using the LEM and FEM methods in Figure 5a to Figure 5b shows similar results as the water diffusion tensor in our study. In spite of the calculating the pixel-dependent scale factor in the VCM method, a higher correlation with water diffusion was observed (Figure 5c). Considering that the local concentration of a charge carrier can affect the linearity between C and D, the CTI method appeared to properly reflect the effects of position-dependent concentration differences by incorporating the actually measured high-frequency conductivity $\sigma_{H}$ into the conductivity tensor image reconstruction. Note that these linear regression analyses should not be considered as a general validity of C=ηD. In CTI method, the mutual restriction of both the ionic and the water mobility by the geometry of the brain medium builds the basis for the relationship of C and D. The voxels in conductivity tensor map are expressed as a sum of products of the carrier concentration, extracellular volume fraction, and the mobility tensor. Since all of the five methods utilized the mobility information embedded in the water diffusion tensor D, the anisotropy ratio of C was primarily determined by the anisotropy ratio of D.

Data acquisition and processing steps are more involved in the CTI method than in those other four methods using DTI. Given that there are various sources of error in the data acquisition and processing stages, subsequent studies are necessary to rigorously validate the performance of the CTI method in terms of errors due to measurement noise and artifacts, partial volume effects, and coregistration of different images. In particular, high-frequency conductivity images using MREPT suffer from errors at boundaries of two regions with different conductivity values and also assumes the piecewise constant conductivity [47], which does not hold in practice. Although the MREPT method we adopted in this paper could reduce such boundary artifacts [14] at the expense of smooth images, future improvements in high-frequency conductivity image reconstruction algorithms are needed to enhance the accuracy of the CTI method in human subjects. The parameter β in (15) was estimated from the literature values and assumed to be constant for all pixels. Although the sensitivity of a reconstructed conductivity tensor image to β appeared to be small [24], clinical studies are needed to find out any practical limitations imposed by this assumption. The CTI method can provide clinically useful information about pathological and physiological changes in cells and cellular structures associated with disease progression. It is one of most promising clinical applications is tumor imaging in terms for early detection, better characterization, and monitoring after a treatment [48,49]. The recovered low-frequency conductivity tensor could be also used to predict internal current pathways and electric field distributions subject to externally injected or induced therapeutic currents in electrical stimulation [28,29].

This study has several limitations that should be considered in the future works. The distribution of conductivity within the giant vesicle phantom does not reflect an adequate anisotropic environment. Future studies should include phantoms mimicking microstructural properties similar to the brain [50]. Diffusion MRI suffers from various systematic errors, such as gradient inhomogeneity [51]. Although we corrected the data using commonly used correction methods, a study of systematic error propagation or noise analysis is needed to enhance the accuracy of the conductivity images. In human brain, conventional DTI measures water diffusion assuming that displacement distribution of water molecules in a given time is a Gaussian function. However, this assumption may not be valid in complex biological tissue where water molecules often show non-Gaussian diffusion [52]. DTI also has limitations in characterizing the diffusion process in areas of low anisotropy and complex fiber structure in a voxel. Future studies with diffusion kurtosis imaging (DKI) or high angular methods (HARDI) can provide better characterization of human brain architecture [52,53]. Cell membranes are assumed to resist low-frequency currents in the CTI approach. As a result, the CTI approach may underestimate the low-frequency conductivity value of tissue including cells with leaky membranes. It would be worthwhile to investigate a more sophisticated CTI model involving such cells.

## 6. Conclusions

In this study, we provided an overview of the current state of the art in MRI-based conductivity tensor reconstruction. The accuracy of five conductivity tensor model was investigated using two phantoms with four electrolytes and two giant vesicles suspensions with known internal conductivity values. The findings show that methods with pixel-dependent scale factors work better than methods with global scale factors. The experimental results showed that the accuracy of the VCM and CTI methods was superior to that of the LEM and FEM methods. Contrary to the four methods using only DTI, the CTI method did not use *prior* information on mean isotropic conductivity values, and produced conductivity images with errors ranging from 1.10% to 5.26%. From in vivo human brain imaging experiments, the reconstructed conductivity values of the white and gray matter using the LEM, VCM, and CTI methods were comparable with the values available in the literature. Except for LEM and FEM, all methods yielded conductivity values of the CSF similar to those of literature. Methods using only the water diffusion tensor and prior knowledge of the isotropic mean conductivity values varied depending on the parameter value used. The CTI method was able to properly handle the effects of different ion concentrations as well as mobilities and extracellular volume fractions in our study. Although the data processing of the CTI method is more involved, it appeared to be the most accurate among the five methods in this study. Future research is required to confirm the clinical utility of these low-frequency conductivity tensor image reconstruction approaches in diagnostic imaging and bioelectromagnetic modeling.

## Figures and Tables

**Table 1:** Compositions of two conductivity phantoms. The electrolyte and giant vesicle suspension are denoted as EL and GVS, respectively. EL${}_{1}$, EL${}_{2}$, and GVS${}_{1}$ were used in phantom #1. EL${}_{3}$, EL${}_{4}$, and GVS${}_{2}$ were used in phantom #2.

Compartment	EL_1_	EL_2_	GVS_1_	EL_3_	EL_4_	GVS_2_
NaCl (g/L)	7.5	3.5	7.5	3	3	3
CuSO${}_{4}$(g/L)	0	1	0	0	0	0
Extracellular	100	100	10	100	100	50
volume fraction						
(%)						
Mobility	high	high	high	low	high	low
σ at 10 Hz (S/m)	1.56	0.83	0.29	0.55	0.70	0.45

**Table 2:** Numbers of pixels in the WM, GM, and CSF ROI of the five human brains. The reconstructed conductivity values in these ROIs were compared based on the five image reconstruction methods and also with existing literature values.

ROI	Subject				
	#1	#2	#3	#4	#5
WM	1093	1026	1257	1070	1047
GM	1057	918	785	927	1023
CSF	209	135	180	237	234

**Table 3:** Errors of the reconstructed conductivity values $\sigma_{L}=\frac{\sigma_{l}+\sigma_{t1}+\sigma_{t2}}{3}$ with respect to the reference values measured by the impedance analyzer at 10 Hz. Note that the reference values were themselves used in the VCM method for the compartments of EL${}_{1}$, EL${}_{2}$, EL${}_{3}$, and EL${}_{4}$.

ROI	LEM (%)	FEM (%)	VCM (%)	CTI (%)
EL${}_{1}$	29.60	86.24	0	1.10
EL${}_{2}$	28.14	75.57	0	4.42
GVS${}_{1}$	131.17	54.83	3.45	1.74
EL${}_{3}$	32.97	73.27	0	3.39
EL${}_{4}$	67.82	66.27	0	5.26
GVS${}_{2}$	28.16	74.22	2.02	2.13

**Table 4:** Mean values of the absolute relative differences ($|rd_{DIR}|$) in the WM, GM, and CSF regions for all five human subjects. The relative differences of the LEM, FEM, VCM, and VFM methods were computed with respect to the CTI method.

Subject	LEM and CTI (%)			FEM and CTI (%)			VCM and CTI (%)			VFM and CTI (%)		
	WM	GM	CSF	WM	GM	CSF	WM	GM	CSF	WM	GM	CSF
#1	44.25	50.99	130.24	63.10	87.90	171.39	50.27	68.60	30.58	91.86	89.73	31.86
#2	49.90	48.29	112.21	58.92	95.20	151.74	53.93	73.25	39.03	91.60	89.08	40.66
#3	40.33	48.87	121.07	63.35	99.29	165.46	51.63	66.47	35.23	92.71	90.61	37.09
#4	43.30	44.00	106.83	49.36	63.41	130.46	49.91	65.17	35.30	92.26	90.56	50.81
#5	46.98	47.66	158.90	56.63	101.28	207.59	62.19	73.10	40.66	89.04	80.15	39.43

## Data Availability

Some of the data used in this study are available on https://iirc.khu.ac.kr/toolbox.html, (accessed on 10 March 2021) or available from corresponding author upon reasonable request.

## Abbreviations

LEM	Linear Eigenvalue Model
FEM	Force Equilibrium Model
VCM	Volume Constraint Model
VFM	Volume Fraction Model
CTI	Conductivity Tensor Imaging
WM	White Matter
GM	Gray Matter
CSF	Cerebrospinal Fluid
MREPT	Magnetic Resonance Electrical Properties Tomography

## Appendix A

In this section, the main symbols used in this paper can be found with description and units.

$\sigma_{L}$	Isotropic low-frequency conductivity (S/m)
$\sigma_{l}$	Longitudinal component of conductivity tensor (S/m)
$\sigma_{t}$	Transversal component of conductivity tensor (S/m)
$d_{l}$	Longitudinal component of water diffusion tensor ( μm2/ms)
$d_{t}$	Transversal component of water diffusion tensor ( μm2/ms)
$\sigma_{iso}$	Isotropic low-frequency conductivity value from the literature (S/m)
$d_{e}$	Isotropic extracellular diffusion coefficient ( μm2/ms)
$D_{e}$	Extracellular water diffusion tensor ( μm2/ms)
